# *Plasmodium falciparum* NIMA-related kinase Pfnek-1: sex specificity and assessment of essentiality for the erythrocytic asexual cycle

**DOI:** 10.1099/mic.0.049023-0

**Published:** 2011-10

**Authors:** Dominique Dorin-Semblat, Sophie Schmitt, Jean-Philippe Semblat, Audrey Sicard, Luc Reininger, Dean Goldring, Shelley Patterson, Neils Quashie, Debopam Chakrabarti, Laurent Meijer, Christian Doerig

**Affiliations:** 1 INSERM-EPFL Joint Laboratory, Global Health Institute, Ecole Polytechnique Fédérale de Lausanne, GHI-SV-EPFL Station 19, CH-1015 Lausanne, Switzerland; 2 INSERM U609, Wellcome Trust Centre for Molecular Parasitology, University of Glasgow, Glasgow G12 8TA, UK; 3 Cell Cycle Group, C.N.R.S., Station Biologique, 29680 Roscoff, Bretagne, France; 4 Biochemistry, University of Kwazulu-Natal, PB X01 Scottsville, South Africa; 5 Department of Molecular Biology and Microbiology, University of Central Florida, 12722 Research Parkway, Orlando, FL 32826, USA; 6 Infection and Immunity, University of Glasgow, 120 University Place, Glasgow G12 8TA, UK

## Abstract

The *Plasmodium falciparum* kinome includes a family of four protein kinases (Pfnek-1 to -4) related to the NIMA (never-in-mitosis) family, members of which play important roles in mitosis and meiosis in eukaryotic cells. Only one of these, Pfnek-1, which we previously characterized at the biochemical level, is expressed in asexual parasites. The other three (Pfnek-2, -3 and -4) are expressed predominantly in gametocytes, and a role for nek-2 and nek-4 in meiosis has been documented. Here we show by reverse genetics that Pfnek-1 is required for completion of the asexual cycle in red blood cells and that its expression in gametocytes in detectable by immunofluorescence in male (but not in female) gametocytes, in contrast with Pfnek-2 and Pfnek-4. This indicates that the function of Pfnek-1 is non-redundant with those of the other members of the Pfnek family and identifies Pfnek-1 as a potential target for antimalarial chemotherapy. A medium-throughput screen of a small-molecule library provides proof of concept that recombinant Pfnek-1 can be used as a target in drug discovery.

## Introduction

Proteins participating in cell signalling, notably the large families of G-protein-coupled receptors and protein kinases (PKs), constitute a vast reservoir of potential molecular targets for chemotherapy in a variety of diseases. PKs are ubiquitous in living organisms, and those of pathogens might represent attractive targets as they offer prospects for selective inhibition. The availability of genomic sequence databases for *Plasmodium falciparum* (Kissinger *et al.*, 2002) has allowed significant progress in our understanding of the complement of molecules involved in cell signalling in malaria parasites, notably through the characterization of the malarial kinome (Anamika *et al.*, 2005; Ward *et al.*, 2004) or potential receptors (Madeira *et al.*, 2008). However, much remains to be elucidated in terms of the function that these elements play in the parasite’s life cycle. Functions in the life cycle have first been established for a few PKs using a gene knockout approach in the *Plasmodium berghei* system (Rangarajan *et al.*, 2005, 2006; Reininger *et al.*, 2005, 2009; Tewari *et al.*, 2005), which is more amenable than *P. falciparum* to genetic manipulation (Carvalho & Ménard, 2005). This culminated in a kinome-wide systematic study identifying all *P. berghei* PKs with a role in transmission (Tewari *et al.*, 2010). In parallel, a number of reverse genetics studies of *P. falciparum* kinases have been published (Abdi *et al.*, 2010; Dorin-Semblat *et al.*, 2007, 2008; Dvorin *et al.*, 2010; Fennell *et al.*, 2009; Halbert *et al.*, 2010; Holland *et al.*, 2009; McRobert *et al.*, 2008; Merckx *et al.*, 2008; Reininger *et al.*, 2009; Taylor *et al.*, 2010). Taken together, these sets of data allow the assignment of roles for specific *Plasmodium* kinases in processes such as parasitaemia growth rate, egress from the erythrocyte, stress response, gametogenesis, meiosis in the mosquito vector and sporozoite infectivity, and have identified a number of kinases as essential for completion of the erythrocytic asexual cycle (reviewed by Doerig *et al.*, 2010).

NIMA-related kinases (*nimA* denotes never-in-mitosis, after the phenotype observed with the *Aspergillus nidulans* mutant that was the first member of the family to be identified), or Neks, constitute a conserved family of enzymes with crucial roles in the regulation of mitosis and meiosis, and are associated with centrosomes, spindle poles and other components of the cell division machinery (Fry *et al.*, 1998; O’Regan *et al.*, 2007; Rhee & Wolgemuth, 1997). Of four Neks represented in the *P. falciparum* kinome, three (Pfnek-2, Pfnek-3 and Pfnek-4) are expressed predominantly in gametocytes; in line with Nek functions in other eukaryotes, the *Plasmodium* nek-2 and nek-4 enzymes were shown to be required for completion of meiosis in the mosquito vector (Reininger *et al.*, 2005, 2009). The only NIMA family member whose mRNA is detectable by microarray throughout the erythrocytic asexual cycle (and also in gametocytes) is Pfnek-1, whose enzymatic properties we described previously using a recombinant enzyme (Dorin *et al.*, 2001). Here we show that an active Pfnek-1 enzyme is indeed expressed in asexual parasites. Furthermore, transgenic parasite lines expressing tagged Pfnek-1 were used to show that the enzyme is sex-specific, with expression detectable only in male gametocytes. We also show that a functional *pfnek-1* gene is required for parasite survival and thus a potential target for chemotherapy, and provide proof of concept that the recombinant enzyme can be used in medium-throughput screening campaigns to identify inhibitors as the first step in the drug discovery process.

## Methods

### 

#### Parasite cultures.

Asexual parasites and gametocytes were cultivated *in vitro* as described previously (Holland *et al.*, 2009). Cultures of asexual parasites were synchronized by using sorbitol treatment (Lambros & Vanderberg, 1979). Gametocytogenesis was induced as described by Carter *et al.* (1993) and gametocytes were maintained until stage V in normal culture media. Stages were monitored by Giemsa staining, and smears were taken for immunofluorescence studies.

#### Transfection constructs.

pCAM-BSD-Nek-1, the plasmid designed for *pfnek-1* gene disruption, was generated by inserting a 557 bp DNA amplicon spanning nucleotides 73–630 of the *pfnek-1* ORF into the pCAM-BSD vector carrying a blasticidine deaminase expression cassette (Sidhu *et al.*, 2005). The insert was obtained by PCR from *P. falciparum* genomic DNA with the following specific primers: forward, 5′-GGGGGATCCAGATTTGGAGAAGTATTTTTAGTA, and reverse, 5′-GGGGCGGCCGCAGGAGACCAATAGTATGGTGT. The primers contained *Bam*HI and *Not*I sites, respectively (underlined), used to insert the PCR product into pCAM-BSD. pCAM-HA-Nek-1, the tagging plasmid, was generated by using the pCAM-BSD-HA vector described previously (Dorin-Semblat *et al.*, 2007) by insertion of an amplicon covering the 3′ end of the *pfnek-1* coding region [nucleotides 2583 to the 3′ end, omitting the stop codon so that the haemagglutinin (HA) epitope was in-frame]. The 768 bp fragment was amplified by PCR from genomic DNA, using the Phusion polymerase, and primers carrying *Pst*I and *Bam*HI restriction sites allowed insertion into the pCAM-BSD-HA vector.

#### Parasite transfection.

Asexual parasites of the 3D7 clone were grown and transfected as described previously (Dorin-Semblat *et al.*, 2007). Briefly, transfection was carried out by electroporation of ring stage parasites with 60 µg plasmid DNA. Blasticidin was added to a final concentration of 2.5 µg ml^−1^ 48 h after transfection. Resistant parasites started to appear 3–4 weeks post-transfection.

#### Genotype analysis.

PCR analysis of transfectants was performed with Ex*Taq* polymerase using genomic DNA with various primer combinations. To detect integration of pCAM-BSD-Nek-1 into the *pfnek-1* locus, the following primers were used: primer 1, 5′-ATGCCAAGTAAATATGATGATGG; primer 2, 5′-TATTCCTAATCATGTAAATCTTAAA; primer 3, 5′-CAATTAACCCTCACTAAAG; primer 4, 5′-GTACTGCTGTTACTATAAC. Primers 1 and 4 correspond to *pfnek-1* sequences, while primers 2 and 3 correspond to pCAM-BSD sequences flanking the insertion site.

To verify the genotype of HA-Pfnek-1-transfected parasites, various combinations of primers were used to test 3′ and 5′ boundaries of the integration site. Primer 1, 5′-GTCAATATAGTAATACTTCAGT; primer 2, 5′-CATGCATGTGCATGCAC; primer 3, 5′-GCCATATTTTATGTAATAATCATGG; primer 4, 5′-GCTTATTTTGTATGATAATATATATAAATAC; primer 5, 5′-CAATTAACCCTCACTAAAG; primer 6, 5′-TATTCCTAATCATGTAAATCTTAAA. Primer 1 is located in the *pfnek-1* sequence lying upstream of the cloned amplicon, primers 3 and 4 hybridize to the *pfnek-13′UTR*, while primers 2, 5 and 6 correspond to pCAM-BSD sequences flanking the insert.

#### Southern blotting.

Genomic DNA was isolated from wild-type parasites and transfectants as described previously (Dorin-Semblat *et al.*, 2007). Genomic DNA (3 µg) was digested with *Spe*I and *Nco*I. Cleaved DNA was separated on a 0.8 % agarose gel, transferred onto a Hybond membrane and probed with the *pfnek-1* fragment used as an insert in the pCAM-BSD-Nek-1 construct. For genotype analysis of HA-tagged transfected parasites, genomic DNA from wild-type 3D7 parasites and transfectants was digested with *Stu*I and *Hin*dIII or *Stu*I and *Bam*HI, and the blot was probed with a 557 bp PCR (nucleotides 577 and 1155 of the *pfnek-1* coding region).

#### Immunofluorescence.

HA-Pfnek-1 protein expression was studied by immunofluorescence assays on cold-methanol-fixed cells. Double labelling experiments were performed as follows: mouse anti-HA antibody (1 : 200 dilution) was incubated with PBS, 1 % BSA, 0.01 % saponin for 1 h. After three washes in PBS, a secondary Alexa Fluor 488 anti-mouse antibody (1 : 1000 dilution) was incubated for 1 hour. Following washes, a rat anti-Pfg377 (1 : 500 dilution) antibody was incubated for 1 h followed by incubation with a secondary Alexa Fluor 594 anti-rat antibody (1 : 1000 dilution). The slides were washed, stained with DAPI for 5 min and mounted with Fluoromount. Labelled specimens were examined with a Zeiss Axioscope microscope in combination with an Orca Digital camera.

Pfnek-1 expression was also investigated using a rabbit immunopurified anti-Pfnek-1 antibody by immunofluorescence assay. For these assays, smears of synchronized stages were fixed with cold acetone for 1 min and blocked in 1 % BSA for 1 h. The labelling was performed for 30 min with the immunopurified antibody and with the pre-immune serum as a control, both diluted 1 : 250 in PBS, 1 % BSA. After two washes, the slides were incubated with an Alexa Fluor 594 anti-rabbit antibody (diluted 1 : 1000) for 30 min. The slides were then washed and mounted with a mounting solution containing DAPI. Image acquisition was performed on a Zeiss Axioplan microscope combined with an Axio Cam HSM B/W camera.

Late-stage gametocytes (stages IV and V) were labelled with antibodies against tubulin and Pf377. Double labelling was performed as above except that a rabbit anti-tubulin antibody (Sigma; 1 : 5000 dilution) and a secondary Alexa Fluor 488 anti-rabbit were used (1 : 1000). Slides were prepared as described previously and visualized with an Olympus Delta Vision microscope IX 71 under a ×100 immersion oil objective.

#### Immunoprecipitation.

Parasite pellets obtained by saponin lysis were sonicated in RIPA buffer (30 mM Tris, pH 8.0, 150 mM NaCl, 20 mM MgCl_2_, 1 mM EDTA, 10 µM ATP, 1 % Triton X-100, 0.5 % Nonidet P-40, 10 mM β-glycerophosphate, 10 mM NaF, 0.1 mM sodium orthovanadate, 1 mM PMSF and Complex protease inhibitors). The lysates were cleared by centrifugation (15 000 r.p.m. for 30 min at 4 °C), and the total amount of proteins in the supernatant was measured using a Bio-Rad protein assay. For immunoprecipitation, parasite extract (300 µg) was incubated on ice for 2 h with pre-immune serum, the immunopurified rabbit anti-Pfnek-1 antibody or anti-HA mouse antibodies. The immunocomplexes were precipitated with 20 µl Protein A-Sepharose CL4B, washed four times in RIPA buffer, once with RIPA buffer supplemented with 0.1 % SDS and once with kinase buffer. Kinase assays were performed on the immunocomplexes using *β*-casein as a substrate.

#### Western blotting.

Protein extracts from synchronized stages (rings, trophozoites and schizonts) were separated on SDS-PAGE gels and transferred onto nitrocelullose membranes as described previously (Dorin *et al.*, 2005). The blots were incubated with an immunopurified chicken antibody (1 : 1000 dilution) or with an immunopurified rabbit antibody (1 : 200 dilution), both directed against a Pfnek-1 peptide (KRGPELPIKGKSKELN), and subsequently incubated with a rabbit anti-chicken or a goat anti-rabbit secondary antibody, respectively, conjugated to peroxidase (1 : 5000 dilution), washed and developed using the ECL detection system. To detect HA-tagged Pfnek-1, protein extracts were prepared and probed as described above. An anti-HA monoclonal peroxidase-conjugated antibody (1 : 1000 dilution, 1 h incubation) was used to detect the HA-tagged kinase, and rabbit serum against anti-PfTrx1 (1 : 4000) was used for a loading control.

#### Kinase assays.

The assays were performed in a standard reaction (30 µl) containing 25 mM Tris/HCl, pH 7.5, 20 mM MgCl_2,_ 2 mM MnCl_2_, 10 µM ATP, 2.5 µCi [γ-^32^P]ATP and 5 µg casein (Sigma). The reactions proceeded for 30 min at 30 °C and were stopped by the addition of Laemmli buffer, boiled for 3 min and analysed by electrophoresis on a 12 % SDS-PAGE gel. The gels were dried and submitted to autoradiography.

#### Medium-throughput screening.

The Pfnek-1 kinase activity was assayed with purified GST-Pfnek-1 [100 ng per well purified as described previously (Dorin *et al.*, 2001)] in 96-well microplates in a standard kinase buffer (20 mM Tris/HCl pH 7.5, 20 mM MgCl_2_, 2 mM MnCl_2_) with β-casein (9 µg) as a substrate, 10 mM β-glycerophosphate, 10 mM sodium fluoride and 15 µM [γ-^33^P]ATP in a final volume of 50 µl. After 30 min incubation at 30 °C, the reaction was stopped by filtration on P81 phosphocellulose paper. Filters were washed in 1 % phosphoric acid and water. Scintillation fluid was added, and incorporation of radioactive phosphate into the substrate was measured in a scintillation counter. Blanks (no kinase) were subtracted from the values. A library comprising 10 480 low-molecular-mass compounds obtained from the World Health Organization was tested on purified Pfnek-1. Five thousand of these compounds were derived from known kinase inhibitory scaffolds. Compounds were first screened at a final concentration of 10 µM. Dose–response curves were performed for all compounds showing more than 50 % inhibition at 10 µM.

## Results and Discussion

### Pfnek-1 protein expression during the erythrocytic asexual cycle

**Fig. 1.**
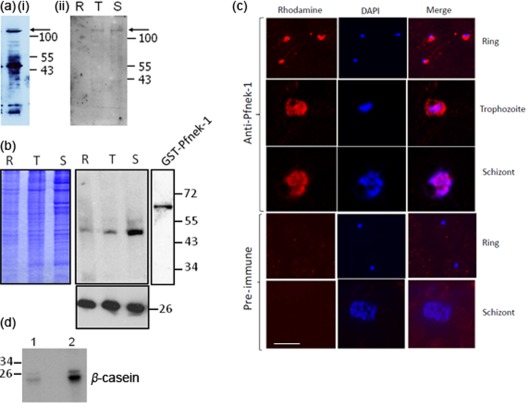
Expression of Pfnek-1 in asexual parasites. (a) Western blot analysis detecting the full-length protein. Asynchronous (i, chicken antibody) or synchronous (ii, rabbit antibody) parasite extract (15 µg) was loaded on the gels. Arrows indicate the position of Pfnek-1. (b) Western blot analysis (rabbit antibody) showing that the antibody recognizes recombinant GST-Pfnek-1 (Dorin *et al.*, 2001) (far right lane) and displays a loading control (antibody against *P. falciparum* 2-Cys peroxiredoxin). R, Rings; T, trophozoites; S, schizonts. Molecular masses of co-migrating markers are indicated in kDa. (c) Immunofluorescence analysis. Synchronized asexual parasites were stained with the immunopurified rabbit anti-Pfnek-1 antibody (top panels). The two bottom panels display negative controls using pre-immune serum. Bar, 5 µm. (d) Immunoprecipitated kinase activity. The anti-Pfnek-1 rabbit antibody was used to immunoprecipitate β-casein kinase activity from 3D7 parasite extracts (lane 2). The pre-immune serum was used as a control (lane 1).

We showed previously that Pfnek-1 transcripts are detectable in asexual parasites as well as in gametocytes (Dorin *et al.*, 2001), consistent with microarray analysis data available on PlasmoDB (Le Roch *et al.*, 2003). However, *Plasmodium* protein expression does not necessarily mirror the presence of the cognate mRNA, and discrepancy between mRNA and protein levels, most often caused by a delay in translation, is not uncommon (Le Roch *et al.*, 2004). To verify that the Pfnek-1 protein is indeed expressed in asexual parasites, rabbit and chicken antibodies were raised against a Pfnek-1-derived peptide and used in Western blot analysis. Both antibodies recognized the catalytic domain of Pfnek-1 expressed in *Escherichia coli* as a GST-fusion protein (Dorin *et al.*, 2001), as well as a >100 kDa band found predominantly in trophozoites and schizonts, which is presumably full-length Pfnek-1 (predicted molecular mass 125 kDa) (Fig. 1a). Additional bands of lower molecular mass were visible, which presumably arise from processing or degradation. In some experiments the >100 kDa band was barely detectable, and the bulk of the signal was provided by a band at 49 kDa with the same expression pattern (Fig. 1b). The size of the band suggests that the C-terminal extension is cleaved in most of the Pfnek-1 molecules, leaving a protein containing essentially only the catalytic domain; additional experiments are required to confirm this possibility.

We used the immuno-purified rabbit anti-Pfnek-1 antibody to study the localization of Pfnek-1 during the parasite asexual cycle. Fluorescent dots were observed near the nuclei at the ring and schizont stages, whereas the pattern was more diffused and appeared as largely cytoplasmic in trophozoites (Fig. 1c). The anti-Pfnek-1 rabbit antibody was able to immunoprecipitate β-casein kinase activity (Fig. 1d), consistent with previously published data demonstrating that the recombinant Pfnek-1 catalytic domain is active against this substrate (Dorin *et al.*, 2001). Pfnek-1 expression and activity in asexual parasites was further confirmed using parasite lines expressing a tagged enzyme (see below).

### A functional *pfnek-1* gene is required for completion of the erythrocytic asexual cycle

**Fig. 2.**
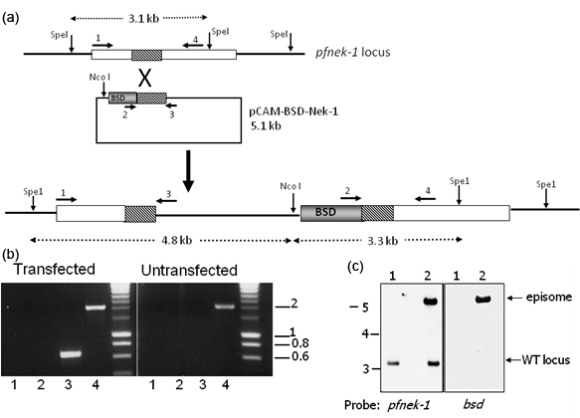
Attempt to disrupt the *pfnek-1* gene. (a) Strategy for *pfnek-1* gene disruption. The knockout vector contains a PCR fragment spanning positions 73–630 of Pfnek-1 coding sequence, excluding two kinase subdomains essential for activity. Single crossover homologous recombination results in a pseudo-diploid configuration with two truncated copies lacking essential catalytic residues. The location of PCR primers (numbered arrows) and the restriction sites used for Southern blot analysis are indicated. (b) PCR genotyping. Genomic DNA was isolated from transfected and parental 3D7 parasites, and subjected to PCR using the indicated primers (see Fig. 2a). Lanes: 1, primers 1+3 (diagnostic for 5′ integration event, 0.6 kb); 2, primers 2+4 (diagnostic for 3′ integration event, 1.5 kb); 3, primers 2+ 3 (diagnostic for the episome, 0.7 kb); 4, primers 1+ 4 (diagnostic for wild-type locus, 2.1 kb). Sizes of co-migrating markers are indicated in kb. (c) Southern blot analysis of transfected parasites. Genomic DNA digested with *Spe*I and *Nco*I was probed with the Pfnek-1 fragment used in the pCAM-BSD-Nek-1 vector (positions 73 to 630 of the coding sequence). Sizes of co-migrating markers are indicated in kb.

To address the question of Pfnek-1 essentiality for completion of the asexual cycle, we followed a strategy used successfully for other *P. falciparum* kinases and which is described in detail elsewhere (Doerig *et al.*, 2010). Briefly, 3D7 parasites were transfected with a plasmid based on the pCAM-BSD vector (Sidhu *et al.*, 2005), in which a central fragment of the Pfnek-1 catalytic domain coding sequence was inserted in such a way that single crossover homologous recombination into the *pfnek-1* locus would result in a pseudo-diploid configuration, in which both truncated copies of the coding sequence lack a subdomain essential for activity (Fig. 2a). The transfected parasites were subjected to blasticidin selection, and the resulting resistant population was examined by PCR for the presence of (i) the wild-type locus, (ii) the episomal vector and (iii) the predicted disrupted locus at both extremities of the inserted plasmid. Three independent transfections were performed, but we did not detect integration even after an extended culture of blasticidin-resistant parasites (up to 6 months); in contrast, we readily succeeded in amplifying the fragment from the wild-type and episomal forms (Fig. 2b). Southern blot analysis was used to verify the integrity of the locus after several months of culture under blasticidin pressure (Fig. 2c).

**Fig. 3.**
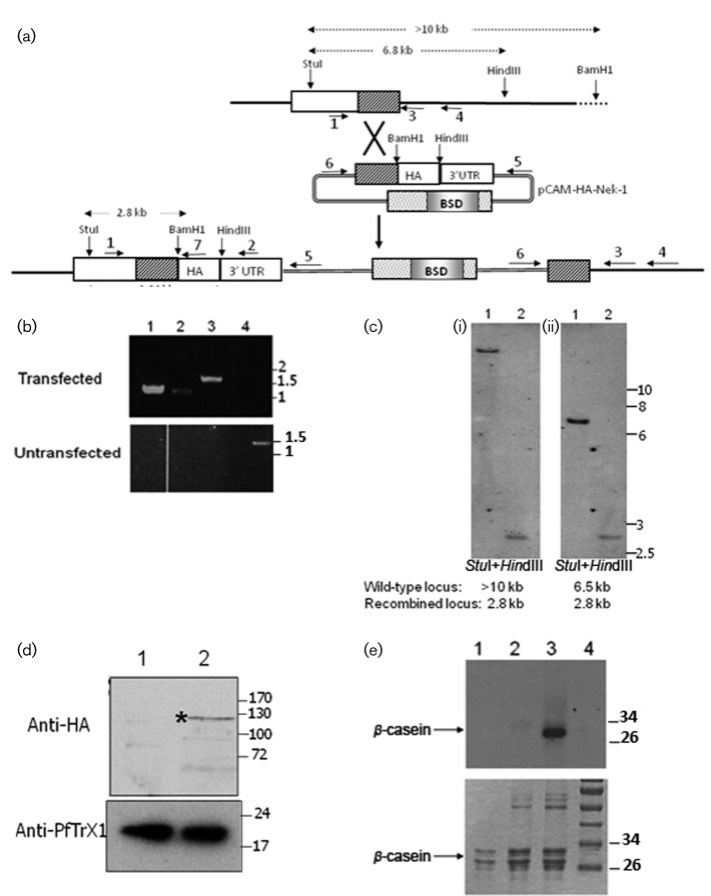
HA tagging of the *pfnek-1* locus. (a) Strategy for HA tagging. The locations of the primers used in PCR diagnostics are indicated with numbered arrows, and the positions of the restriction sites used for Southern blot analysis are shown. (b) PCR genotyping analysis. Genomic DNA was isolated from pCAM-HA-nek-1-transfected parasites and from parental 3D7 parasites, and subjected to PCR using the indicated primers (see Fig. 3a). Lanes: 1, primers 1+7 (diagnostic for 5′ integration event, 1.2 kb); 2, primers 6+3 (diagnostic for 3′ integration event, 1.2 kb); 3, primers 6+4 (diagnostic for 3′ integration event, 1.5 kb); 4, primers 1+4 (diagnostic for wild-type locus, 1.5 kb). Sizes of co-migrating markers are indicated in kb. (c) Southern blot analysis of transfected parasites. Total DNA digested with *Stu*I and *Hin*dIII (i) or *Stu*I and *Bam*HI (ii) was probed with a fragment spanning nucleotides 577–1155 of the *pfnek-1* coding region. Sizes of co-migrating markers are indicated in kb. (d) Western blot analysis of HA-tagged Pfnek-1. Protein extracts (15 µg) from the parental 3D7 wild-type clone (lane 1) and pCAM-HA-nek-1-transfected parasites (lane 2) were probed with an anti-HA antibody. Sizes of co-migrating markers are indicated in kDa. (e) Anti-HA antibodies immunoprecipitate kinase activity from transfected parasites. Anti-HA antibodies were incubated with extracts from the parental 3D7 wild-type clone (lane 2) or pCAM-HA-Nek-1-transfected parasites (lane 3). Immunoprecipitated material was assayed for β-casein activity. Lane 1 shows the kinase assay with β-casein alone (no immunoprecipitate added), and the molecular mass marker is shown in lane 4. Molecular masses are indicated in kDa.

There is still the possibility at this stage that the absence of integration was due to the lack of potential of the *pfnek-1* locus to recombine. To investigate this possibility, we transfected parasites with pCAM-HA-Nek-1, a pCAM-BSD plasmid containing the 3′ end of the Pfnek-1 coding region fused to an HA epitope, followed by the 3′ untranslated region from the *P. berghei dhfr-ts* gene. Recombination with the genomic locus is expected to result in the formation of a functional locus expressing an HA-tagged Pfnek-1 protein (plus an inactive truncated copy) (Fig. 3a). PCR (Fig. 3b) and Southern blot (Fig. 3c) analyses conducted 8 weeks post-transfection showed that integration had occurred (and had been detected in three of three transfected populations as early as 5 weeks post-transfection by nested PCR), demonstrating that the locus can be readily modified if no loss of function is incurred. The 6.8 and >10 kb wild-type bands on the Southern blot (Fig. 3c, lanes 1) are undetectable in the (uncloned) transfected population; likewise, amplicons diagnostic for the wild-type locus were not detected by PCR. This indicates that parasites with a modified locus have overgrown those with a wild-type locus and containing the episome, presumably because in the former subpopulation (but not in the latter) all daughter merozoites contain the resistance cassette. An anti-HA antibody did not give any detectable signal from mock-transfected parasites in Western blot analysis, but extracts from the transfected population yielded a signal at the expected size of 125 kDa (Fig. 3d). Immunoprecipitates obtained from transfected parasite extracts (but not from extracts from the wild-type parental clone) with the anti-HA antibody contained casein kinase activity, further confirming that Pfnek-1 HA-tagging occurred and did not interfere with enzymic function. Taken together, our transfection data showing that the *pfnek-1* locus can be modified only if the change does not cause loss of function demonstrate that Pfnek-1 plays an important role in the erythrocytic asexual cycle.

### Sex specificity of Pfnek-1 expression

**Fig. 4.**
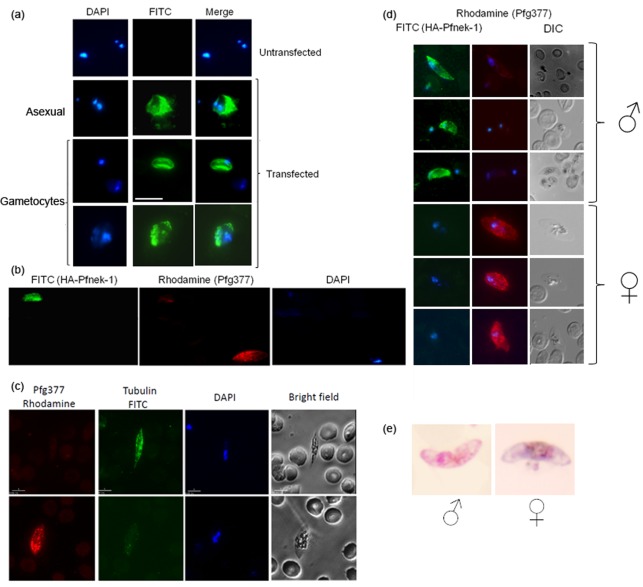
Immunofluorescence analyses of male and female gametocytes. (a) Detection of HA-Pfnek-1 in asexual parasites (line 2) and gametocytes (lines 3 and 4) transfected with pCAM-HA-Nek-1 using an anti-HA antibody. The top line shows the negative control (staining of wild-type parasites with the anti-HA antibody). Bar, 10 µm. (b) A single microscopic field is presented, showing mutually exclusive staining with both antibodies. DAPI (blue), and anti-mouse–FITC (green) and anti-rat–rhodamine (red) secondary antibodies were used. (c) Tubulin is expressed predominantly in male gametocytes. Co-staining was performed with the α-tubulin antibody and an antibody against Pfg377, a protein expressed predominantly in female gametocytes. (d) Further examples of the sex-specificity of HA-Pfnek-1 expression. (e) Typical images of Giemsa-stained stage V gametocytes, used to determine the sex ratio in Table 1. Bar, 10 µm. ♂, Male; ♀, female.

The availability of parasite populations expressing HA-tagged Pfnek-1 allowed protein expression to be monitored throughout parasite development. Immunofluorescence analysis (IFA) using anti-HA antibodies confirmed the data obtained with the anti-Pfnek-1 antibody (Fig. 1), showing expression in asexual parasites, and revealed expression in gametocytes as well (Fig. 4a). In gametocytes, staining seems to concentrate in an elongated structure on one side of the cell; in some instances FITC labelling seems to accumulate at the gametocyte poles. This is in contrast with Pfnek-2, which labels clear microtubular structures in female gametocytes (Reininger *et al.*, 2009). Interestingly, we noticed that only a small fraction (approx. 5 %, Table 1) of the gametocytes yielded an HA signal, suggesting that expression of Pfnek-1 might be restricted to male gametocytes. To test this hypothesis, we performed co-IFA of gametocyte cultures with a mouse anti-HA antibodies and a rat antibody directed against Pfg377, a protein that is associated with osmiophilic bodies and is preferentially expressed in female gametocytes (Alano *et al.*, 1995; de Koning-Ward *et al.*, 2008), using anti-mouse and anti-rat secondary antibodies conjugated to FITC (green) and rhodamine (red), respectively. The outcome of this experiment was clear: Pfg377-positive (red) gametocytes did not display any FITC (green) staining, and the FITC-positive cells were only very faintly stained with the Pfg377 antibody (Fig. 4b). Fig. 4(c) presents two gametocytes found in the same microscopic field and illustrates the largely mutually exclusive green–red staining. Additional representative examples are shown in Fig. 4(d). We found that approximately 95 % of the gametocytes gave a strong Pfg377 (red) signal, while the remaining 5 % showed evidence of HA-Pfnek-1 expression (green) (Table 1). This is very similar to the sex ratio that we measured in cultures of our parental wild-type 3D7 parasites using either Giemsa staining, which allows sexing of mature gametocytes (Schwank *et al.*, 2010) (Fig. 4e and Table 1), or staining with anti-α-tubulin antibodies, which was shown to react specifically with male gametocytes in late stages (IV and V) of gametocytogenesis (Fig. 4c and Table 1). Taken together, these data demonstrate (i) that Pfnek-1 is expressed only in male gametocytes, in line with the conclusion reached by Khan and co-workers about the *P. berghei* orthologue of Pfnek-1 on the basis of mass spectrometry analysis of purified male or female gametocyte populations (Khan *et al.*, 2005), and (ii) that the C-terminal HA tag did not affect the sex ratio and hence, presumably, the function of Pfnek-1 in male gametocytes.

**Table 1.** Sex ratio in gametocyte cultures, determined by using various methods Male and female gametocytes were counted on smears of the following stages: stages IV and V for labelling of HA-Pfnek-1 transgenic parasites with antibodies against HA and Pfg377; stage V for Giemsa-stained gametocytes of the wild-type 3D7 clone; stages IV and V for labelling gametocytes of the wild-type 3D7 clone with antibodies against α-tubulin and Pfg377. See text for details.

	No. of female gametocytes	No. of male gametocytes	Sex ratio (%)*
Pfg377/HaNek1	155 (red)	8 (green)	5
Giemsa stain	117 (blue)	8 (pink)	6.4
Pfg377/Tubulin	235 (red)	21 (green)	8

* Male/(male+female)×100.

### Potential for antimalarial drug discovery

Our reverse genetics data validate Pfnek-1 as a potential drug target for antimalarial chemotherapy. To provide a proof-of-concept that the recombinant protein can be used in screening operations, we performed a medium-throughput screen of a library of small molecules on recombinant GST-Pfnek-1 (see Methods). Of 10 480 compounds tested, 23 showed more than 50 % inhibition at 10 µM. This low hit rate (0.22 %) illustrates the specificity of the Pfnek-1 inhibition assay. IC_50_ values were determined and ranged from 0.85 to 10 µM (data not shown; detailed results from the screen and post-screen hit characterization are outside the scope of this study and will be published elsewhere). This indicates that the activity of the recombinant Pfnek-1 catalytic domain is sufficient to run screening programs, extending smaller-scale studies where compounds from natural sources were found to inhibit recombinant Pfnek-1 (Desoubzdanne *et al.*, 2008; Laurent *et al.*, 2006). A promising avenue to follow would be to screen on Pfnek-1 (and other *P. falciparum* kinases) the so-called ‘malaria boxes’, collections of compounds identified in cellular screens as possessing selective parasiticidal activity (Gamo *et al.*, 2010; Guiguemde *et al.*, 2010; Plouffe *et al.*, 2008). Interestingly, Pfnek-1 was predicted on a chemoinformatics basis to be a target of the some of the hit compounds in the malaria box from the GlaxoSmithKline study (Gamo *et al.*, 2010).

Since Pfnek-1 is now known to be expressed in male gametocytes and Pfnek-2 and -4 in female gametocytes (and since the meiosis-related phenotypes of both Pbnek-2 and -4 are carried by female gametocytes/gametes), interference with Nek function represents a potential avenue for transmission blocking as well as curative intervention. Because of the relatedness of these enzymes to each other, inhibitors affecting more than one enzyme of the family might conceivably be identified. The benefits of multi-target strategies for treatment with kinase inhibitors have been discussed in other contexts (for example, see Sathornsumetee & Reardon, 2009). The Pfnek-1, -2 and -4 recombinant enzymes all share the properties that are required for screening operations (Dorin *et al.*, 2001; Reininger *et al.*, 2005, 2009). Thus, the NIMA-related kinases represent genetically validated targets for a multi-pronged approach against multiple stages of the parasite life cycle.
